# Development of a Multiprotein Classifier for the Detection of Early Stage Ovarian Cancer

**DOI:** 10.3390/cancers14133077

**Published:** 2022-06-23

**Authors:** Kristin L. M. Boylan, Ashley Petersen, Timothy K. Starr, Xuan Pu, Melissa A. Geller, Robert C. Bast, Karen H. Lu, Ugo Cavallaro, Denise C. Connolly, Kevin M. Elias, Daniel W. Cramer, Tanja Pejovic, Amy P. N. Skubitz

**Affiliations:** 1 Department of Laboratory Medicine and Pathology, University of Minnesota, Minneapolis, MN 55455, USA; boyla002@umn.edu; 2 Division of Biostatistics, University of Minnesota, Minneapolis, MN 55455, USA; pete6459@umn.edu; 3 Department of Obstetrics, Gynecology and Women’s Health, University of Minnesota, Minneapolis, MN 55455, USA; star0044@umn.edu (T.K.S.); gelle005@umn.edu (M.A.G.); 4 Department of Outcomes Research, Cleveland Clinic, Cleveland, OH 44195, USA; pux@ccf.org; 5 Department of Quantitative Health Sciences, Cleveland Clinic, Cleveland, OH 44195, USA; 6 Department of Experimental Therapeutics, The University of Texas M. D. Anderson Cancer Center, Houston, TX 77030, USA; rbast@mdanderson.org; 7 Department of Gynecological Oncology and Reproductive Medicine, The University of Texas M. D. Anderson Cancer Center, Houston, TX 77030, USA; khlu@mdanderson.org; 8 Unit of Gynecological Oncology Research, European Institute of Oncology IRCCS, 20139 Milano, Italy; ugo.cavallaro@ieo.it; 9 Fox Chase Cancer Center, Philadelphia, PA 19111, USA; denise.connolly@fccc.edu; 10 Division of Gynecologic Oncology, Department of Obstetrics and Gynecology and Reproductive Biology, Brigham and Women’s Hospital, Dana-Farber Cancer Institute, Harvard Medical School, Boston, MA 02115, USA; kelias@bwh.harvard.edu; 11 Department of Obstetrics, Gynecology and Reproductive Biology, Brigham and Women’s Hospital, Harvard Medical School, Boston, MA 02115, USA; dcramer@bwh.harvard.edu; 12 Department of Obstetrics and Gynecology, Oregon Health & Science University, Portland, OR 97239, USA; pejovict@ohsu.edu; 13 Knight Cancer Institute, Oregon Health & Science University, Portland, OR 97239, USA

**Keywords:** ovarian cancer, protein biomarkers, early detection

## Abstract

**Simple Summary:**

When ovarian cancer is detected early, the survival rate is high. Unfortunately, existing blood tests are neither sensitive nor specific enough to screen women for ovarian cancer. The purpose of this study was to determine the levels of 92 cancer-related proteins in the blood of women with ovarian cancer compared to healthy women in order to develop a test for ovarian cancer detection. We tested the blood of more than 400 women and identified four proteins that, when combined, successfully detected over 90% of the women with ovarian cancer. We then tested more than 700 additional blood samples and found that the combination of the four proteins successfully distinguished the majority of the blood samples from women with both early and late stages of ovarian cancer compared to healthy women. These four proteins show promise in the development of a test to detect the early stages of ovarian cancer.

**Abstract:**

Background: Individual serum biomarkers are neither adequately sensitive nor specific for use in screening the general population for ovarian cancer. The purpose of this study was to develop a multiprotein classifier to detect the early stages of ovarian cancer, when it is most treatable. Methods: The Olink Proseek Multiplex Oncology II panel was used to simultaneously quantify the expression levels of 92 cancer-related proteins in sera. Results: In the discovery phase, we generated a multiprotein classifier that included CA125, HE4, ITGAV, and SEZ6L, based on an analysis of sera from 116 women with early stage ovarian cancer and 336 age-matched healthy women. CA125 alone achieved a sensitivity of 87.9% at a specificity of 95%, while the multiprotein classifier resulted in an increased sensitivity of 91.4%, while holding the specificity fixed at 95%. The performance of the multiprotein classifier was validated in a second cohort comprised of 192 women with early stage ovarian cancer and 467 age-matched healthy women. The sensitivity at 95% specificity increased from 74.5% (CA125 alone) to 79.2% with the multiprotein classifier. In addition, the multiprotein classifier had a sensitivity of 95.1% at 98% specificity for late stage ovarian cancer samples and correctly classified 80.5% of the benign samples using the 98% specificity cutpoint. Conclusions: The inclusion of the proteins HE4, ITGAV, and SEZ6L improved the sensitivity and specificity of CA125 alone for the detection of early stages of ovarian cancer in serum samples. Furthermore, we identified several proteins that may be novel biomarkers of early stage ovarian cancer.

## 1. Introduction

Ovarian cancer is the fifth leading cause of cancer deaths in women in the United States. In 2022, nearly 20,000 women will be diagnosed with ovarian cancer and more than 12,000 will die of their disease [1]. Due to vague symptoms and lack of adequate screening tests, most women are not diagnosed with ovarian cancer until it is advanced, and the 5 year survival rate is ~30% [2]. In contrast, for women diagnosed with stage I ovarian cancer, limited to the ovaries, the long-term survival rate is almost 90%, and for stage II, limited to the pelvis, the survival rate is 70% [2], highlighting the need for strategies for earlier detection. 

CA125, the most well-known ovarian cancer biomarker, is not expressed in ~20% of ovarian cancers [3,4], and therefore is not adequately sensitive to screen the general population for ovarian cancer. It has been suggested that the addition of biomarkers complementary to CA125 may increase the sensitivity of detecting early stage disease. In addition to proteins, other molecules have been explored as potential biomarkers for ovarian cancer. Autoantibodies against cancer antigens such as TP53 have been identified in 20–30% of ovarian cancer cases tested and may provide additional lead time over CA125 [5]. Serum microRNAs have been identified as candidate ovarian cancer biomarkers [6,7], and circulating tumor DNA has also been tested as a method for early detection [8]. 

New technology has been developed that makes it possible to measure levels of multiple protein biomarkers simultaneously in very small volumes of serum or plasma. The proximity extension assay (PEA) [9,10] technology from Olink Bioscience permits the simultaneous quantification of 92 disease-related protein biomarkers, using sample volumes as low as 1 µL. PEA is an innovative technology that combines the specificity of antibody-based detection methods with the sensitivity of PCR, allowing multiplex biomarker quantification with high precision. In a feasibility study, we tested a small set of early stage serous ovarian cancer, late stage serous ovarian cancer, benign ovarian tumors, and healthy controls (20 of each) on the Proseek Oncology Iv2 panel to identify candidate biomarkers for early stage serous ovarian cancer [11]. We demonstrated that the Proseek assay panels provide similar results to conventional assays for CA125 and can be used to identify new candidate biomarkers that, when combined with CA125, can improve the detection of ovarian cancer over using CA125 alone. From the Proseek Oncology Iv2 data, we developed a 12-protein classifier with improved sensitivity over CA125 alone when comparing sera from healthy women with early stage ovarian cancer patients. Since the Oncology Iv2 panel is no longer commercially available, further discovery phase experiments are now carried out using the updated Oncology II panel. To confirm our findings with the earlier version, we used the Proseek Oncology II panel to quantify the expression of 92 proteins in sera from women with late-stage high-grade serous ovarian cancer, and identified a multiprotein classifier that could distinguish late stage ovarian cancer from age-matched healthy control samples [12]. 

In this study, we measured protein levels in sera collected at two different institutions from women diagnosed with early stage ovarian cancer (all subtypes) using the Proseek Oncology II panel. Using these data, we developed a multiprotein classifier that could discriminate between early stage ovarian cancer and healthy controls. We then used a second cohort of serum samples collected from women at four different institutions to validate our classifier and determine its predictive value using sera from women with late stage ovarian cancer and benign ovarian conditions.

## 2. Materials and Methods

### 2.1. Serum Samples 

Blood samples were collected prior to treatment (surgery or chemotherapy) from women diagnosed with stage I–II epithelial ovarian cancer of all subtypes, benign ovarian conditions, or age-matched healthy controls under IRB-approved protocols. Cohort #1 samples were collected at the University of Minnesota (Minneapolis, MN, USA) and the M.D. Anderson Cancer Center (Houston, TX, USA); these samples were used in the discovery phase of experiments to develop a multiprotein classifier. Cohort #2 samples were collected at the Brigham Women’s Hospital, Harvard Medical School (Boston, MA, USA), Fox Chase Cancer Center (Philadelphia, PA, USA), European Institute of Oncology (Milan, Italy), and Oregon Health & Science University (Portland, OR, USA). These samples were used for the validation phase of the experiments. 

### 2.2. Olink Proseek Oncology II Assay 

The levels of 92 oncology related proteins were quantified in 1 ul of serum using the Proseek Oncology II Proximity Extension Immunoassay Panel (Olink, Uppsala, Sweden) as previously described [12]. Samples were randomly assigned to 96-well plates using stratified randomization based on the institution of origin, diagnosis (healthy vs. cancer), ovarian cancer subtype, age, and race (when available). Samples were run on the Proseek Oncology II panel to quantify the level of protein expression. Each sample was mixed with the Proseek Oncology II reagents according to the manufacturer’s instructions and quantified by Q-PCR using a Fluidigm^®^ BioMark™ HD high-throughput PCR instrument at the University of Minnesota Genomics Center. The Proseek platform includes three “interplate controls” for data normalization between plates and three “negative controls” to establish background levels. Internal controls for incubation and extension are included by Olink in each assay for quality control. The Proseek^®^ assay reports relative quantification on a log2 scale, as Normalized Protein eXpression (NPX) values, which was normalized according to the manufacturer’s protocols. Samples that did not pass Olink quality control were not included in the analysis. 

### 2.3. Identification of Unstable Proteins

In Cohort #1, case-control differences between institutions were explored by fitting a linear regression model with an interaction term of institution (MN vs. TX) and disease status (ovarian cancer vs. healthy), along with the corresponding main effects, for each protein with a Holm’s adjustment to account for multiple testing. Proteins whose case-control differences varied significantly between institutions were excluded due to concern that these proteins’ levels were unstable, meaning too sensitive to preanalytical conditions (e.g., environmental factors such as preprocessing storage time or the pre- or postcentrifugation temperatures). We compared our findings from Cohort #1 to previous work that investigated the impact of environmental factors on quantified protein levels for Olink panels for cardiovascular disease (Olink CVD I) and inflammation (Olink Inflammation I) [13]. 

### 2.4. Data Normalization for Cohort #2

Twenty-two “bridge” samples (12 ovarian cancer and 10 healthy controls; 2–3 samples per 96-well plate) were used to normalize data between Cohort #1 and Cohort #2 using Olink’s recommended approach [14]. Specifically, differences in NPX values were calculated between the “bridge” samples from Cohort #1 and Cohort #2, and then the median of these pairwise differences were calculated for each protein, which we call the normalization factor. The NPX values for each of the proteins for samples in Cohort #2 were normalized by subtracting the protein-specific normalization factor. This data normalization is necessary since the Proseek assay reports relative (vs. absolute) quantification.

### 2.5. Statistical Analysis

The data were normalized by the University of Minnesota Genomics Center, per the manufacturer’s protocol [15]. Differences in mean expression between cancer and healthy samples were determined using two-sample *t*-tests assuming unequal variances with *p*-values adjusted to control the false discovery rate at 5%. Single-protein classification accuracy was evaluated using the empirical receiver operating characteristic (ROC) curve and was summarized by the area under the ROC curve (AUC) and the sensitivities corresponding to specificities of 0.95 and 0.98 (i.e., ROC (0.05) and ROC (0.02), respectively). To summarize the value added beyond the contribution of CA125, the same summaries (AUC, ROC (0.05), and ROC (0.02)) were calculated for two-protein classifiers that included CA125 and one other protein. These two-protein classifiers were fit on Cohort #2 using a method [16] to maximize the sensitivity for a fixed specificity of 0.95 and assessed on Cohort #1. Confidence intervals (CIs) for AUC, ROC (0.05), and ROC (0.02) were calculated using a nonparametric bootstrap approach.

A multiprotein classifier was developed to differentiate healthy controls from early stage ovarian cancer cases using least absolute shrinkage and selection operator (LASSO) logistic regression with the tuning parameter chosen using 10-fold cross-validation to be that with a cross-validation error within 1 standard error of the minimum cross-validation error (“lambda.1se”) [17]. Summaries of the classification accuracy were estimated using the predicted probabilities from the held-out cross-validation folds. To obtain CIs for AUC, ROC (0.05), and ROC (0.02) for the multiprotein classifier, the bias-corrected bootstrap case cross-validation method of Jiang et al. [18] was used. The difference in AUCs between different classifiers was tested using a bootstrap method for correlated ROC curves. All analyses were performed in R version 4.0.2 (R Foundation for Statistical Computing, Vienna, Austria) using the R packages glmnet [19], maxTPR [20], and pROC [21].

### 2.6. Unsupervised Hierarchical Clustering Analysis

Unsupervised clustering methods were applied to the data to identify clusters of proteins and visually evaluate their association with disease status. Unsupervised hierarchical clustering (uncentered correlation using centroid linkage) was completed using Cluster 3.0 [22] and visualized using Treeview (v1.1.6r4; Ref [23]). Principal component analysis (PCA) was performed using the prcomp function in R [24] and t-distributed Stochastic Neighbor Embedding (t-SNE) was done using the Rtsne package in R [25].

## 3. Results

### 3.1. Cohort #1 Demographics

The Proseek Oncology II panel was used to quantify the expression of 92 cancer-related proteins in 1 μL of serum from 336 healthy women and 116 women with early stage ovarian cancer from the University of Minnesota and the MD Anderson Cancer Center (Cohort #1; Table 1). The ovarian cancer samples were comprised of the major epithelial subtypes of ovarian cancer, with almost half of the samples from women diagnosed with high-grade serous ovarian cancer (HGSOC; 46%). The remaining ovarian cancer samples were from women with endometrioid (18%), mucinous (13%), clear cell (12%), or with mixed ovarian cancer subtypes (11%).

### 3.2. Identification of Unstable Proteins

**Figure 1:**
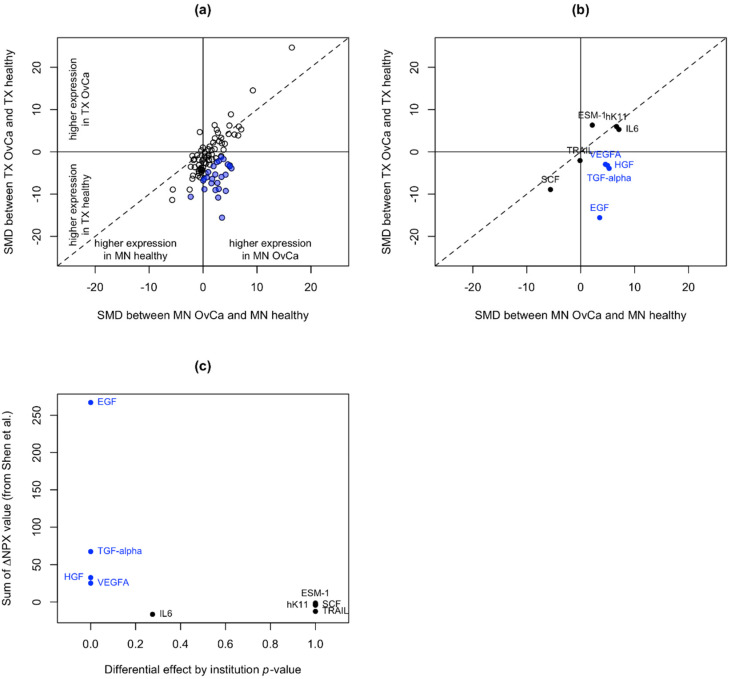
Defining proteins that may be sensitive to preanalytical variation. (**a**) The standardized mean differences (SMDs, i.e., *t*-statistics) in protein levels between those with and without cancer were compared for Texas (TX) vs. Minnesota (MN). Blue points are proteins that were significantly differentially expressed between institutions (e.g., overexpressed in MN cancers vs. underexpressed in TX cancers). (**b**) The same plot as in (**a**), except only the proteins tested to determine their preanalytical variation in Shen et al. [13] are plotted. (**c**) Shen et al.’s measurement of protein instability (sum of ∆NPX values; higher values indicate more instability) was compared to the *p*-values for differential effects by institution, where small *p*-values are evidence of instability. The differential effect by institution *p*-values for all differentially expressed proteins are in Table S1, along with the mean differences by institution.

The Proseek assay uses PEA technology in which oligonucleotide-labeled antibody pairs are used to quantify proteins by real-time PCR. To determine whether any of the protein measurements may have been sensitive to preanalytical variation during the sample collection or processing, we compared the standardized mean differences (SMDs) in protein levels between subjects with and without cancer for samples from MD Anderson (TX) and the University of Minnesota (MN) (Figure 1a). Twenty-four proteins were significantly differentially expressed between institutions (e.g., overexpressed in MN cancer samples and underexpressed in TX cancer samples; Figure 1a, blue circles). These findings are consistent with previous work by Shen et al. [13], who examined the effects of preanalytical variables on proteins quantified using the Proseek Cardiovascular Disease I and Inflammation I assay panels. The SMDs in the protein levels for the nine proteins in our study, which were also examined in Shen et al.’s study, are shown in Figure 1b. When the protein instability measurement from Shen et al. (sum of ΔNPX values, with higher values indicating more instability) was compared to the *p*-values for the differential effects by institution in our study (where small *p*-values are evidence of instability), all four of the proteins with significant *p*-values for the differential effects by institution (blue filled circles in Figure 1b) also had the highest sum of the ΔNPX values (blue circles in Figure 1c), consistent with our hypothesis that these proteins are unstable and sensitive to preanalytical variation. Therefore, the 24 proteins that were significantly differentially expressed between TX and MN were removed from downstream analysis, due to concern that they would not serve well in the development of future clinical biomarker panels. The list of 24 proteins and the mean differences by institution can be found in Table S1.

### 3.3. Identification of Candidate Biomarkers for Early Stage Ovarian Cancer

In our previous study, the Proseek Oncology II assay measurements for both CA125 and HE4 correlated with the clinical values or ELISA measurements in serum samples obtained from women with late-stage high-grade serous ovarian cancer [12]. In this study, we made a similar comparison comparing the Proseek NPX values with the clinical values or ELISA measurements for CA125. Again, we found the measurements were highly correlated (r = 0.83). 

**Figure 2:**
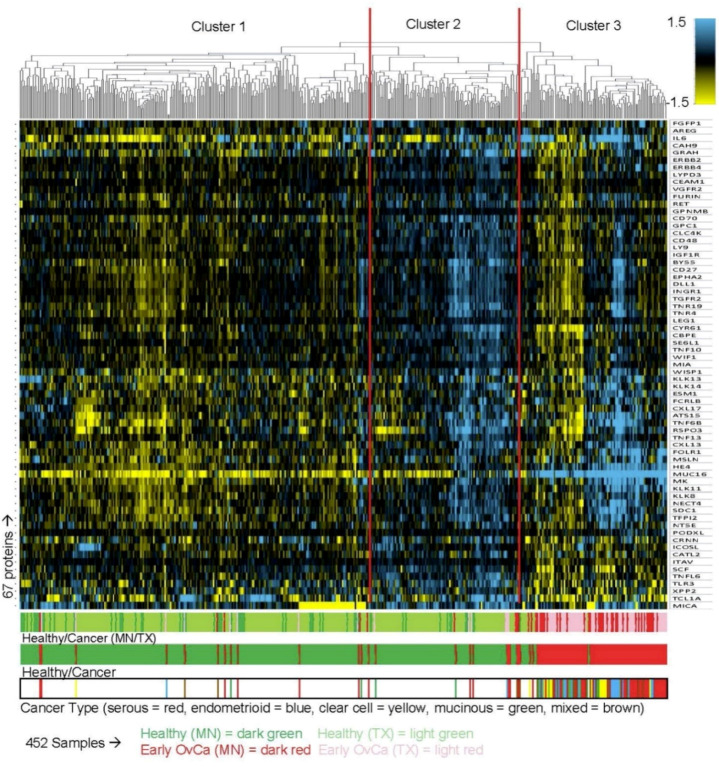
Hierarchical clustering of 452 serum samples from Cohort #1 based on 67 proteins (excluding FOLR3). Blue indicates proteins with high levels of expression, while yellow indicates proteins with low levels of expression. Three major clusters were identified. The three color bands at the bottom identify the samples. Band #1, sample type and institution: Light red, 70 early stage ovarian cancer (TX); dark red, 46 early stage ovarian cancer (MN); light green, 275 healthy (TX); dark green, 61 healthy (MN). Band #2, overall sample type: 116 cancer (red) and 336 healthy (green). Band #3, ovarian cancer subtypes: 53 serous (red), 21 endometrioid (blue), 14 clear cell (yellow), 15 mucinous (green), and 13 mixed (brown).

We also performed unsupervised clustering of the 452 samples in Cohort #1 based on 67 proteins to visualize the protein expression differences between the samples from the two institutions. Plotting of the first three principal components (Figure S1a) and t-SNE plots (Figure S1b) show that the early stage ovarian cancer serum samples separate from the healthy serum samples regardless of the institution of origin. The protein folate receptor-gamma (FR-gamma/FOLR3) was not included in these analyses because it was expressed at high levels in a subset of serum samples that did not correlate with ovarian cancer status. Similar to the PCA and t-SNE analysis, unsupervised hierarchical clustering identified major clusters of cancer vs. healthy (Figure 2). Clusters 1 and 2 are composed primarily of samples from healthy controls, while cluster 3 is primarily from ovarian cancer samples, with some exceptions scattered throughout. The hierarchical clustering revealed a group of proteins that have previously been reported to be expressed at elevated levels in ovarian cancer serum samples, including: CA125 (MUC16), HE4, MSLN, MK, KLK8, KLK11, NECT4, FOLR1, as well as KLK13, KLK14, and IL6 [3,26,27,28,29,30,31,32,33,34,35]. The two clusters formed by samples from healthy individuals are divided by a general upregulation vs. downregulation of all proteins. There is no evidence of batch effect between the sources, as samples from both institutions are interspersed. 

**Figure 3:**
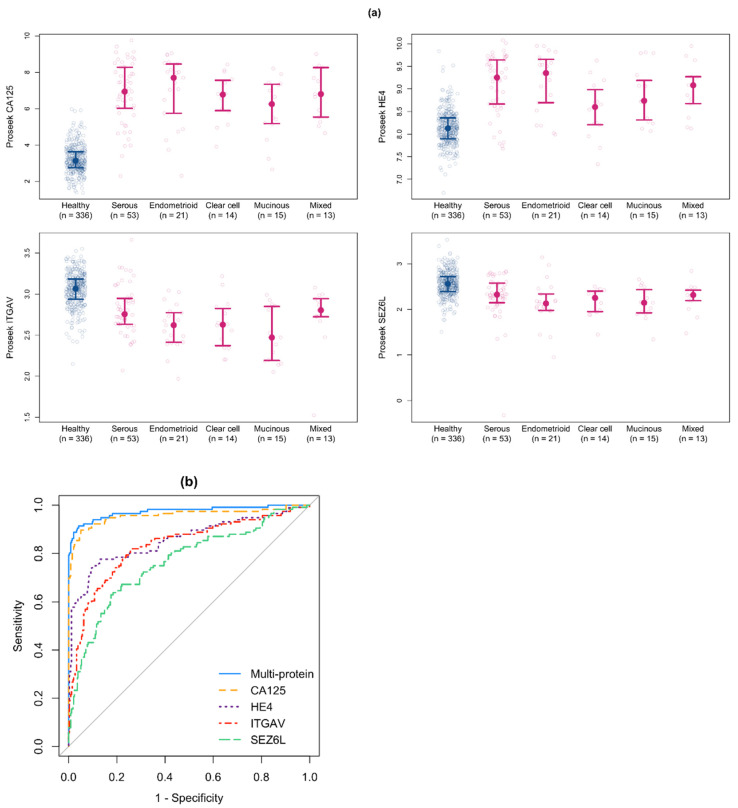
Development of a multiprotein classifier from samples in Cohort #1. (**a**) Proseek Oncology II NPX values were plotted with the median and 25th and 75th percentiles for the healthy patients (blue) and the 5 subtypes of early stage ovarian cancer (pink) for the four proteins included in the multiprotein classifier. (**b**) ROC curves for the multiprotein classifier and each of the four individual proteins included in the multiprotein classifier.

By FDR-adjusted two-sample *t*-tests, the mean levels of 38 of the 68 proteins differed significantly (*p* < 0.05) between the early stage (I-II) ovarian cancer and healthy samples (Table 2); seventeen of these proteins were elevated in the ovarian cancer samples compared to the healthy control samples, including CA125 and HE4. The Proseek NPX values for CA125 and HE4 were elevated in ovarian cancer samples from all subtypes (Figure 3a). The mean NPX expression values by diagnosis of all 68 proteins is provided in Table S2.

To summarize the sensitivity (true positive rate; the probability that an ovarian cancer specimen will be correctly identified as cancer) and specificity (true negative rate; the probability that a healthy control sample will be correctly identified as healthy) of each protein individually across all classification thresholds, we calculated the AUC for each of the 68 proteins. In total, 11 proteins had an estimated AUC of >0.70 (Table 3). Seven of these eleven proteins were elevated in the early stage (I–II) ovarian cancer samples compared to control samples, while ITGAV, SCF, SEZ6L, and FASLG were decreased. As expected, CA125 had the highest AUC (0.958, 95% CI: 0.928–0.982) and HE4 was second with an AUC of 0.857 (95% CI: 0.808–0.901). However, when the AUCs for the individual proteins in combination with CA125 were considered, HE4 was outperformed by multiple other proteins. The AUC values and sensitivity at 95% and 98% specificity for all 68 proteins individually and in combination with CA125 are listed in Table S3.

The sensitivity at two fixed levels of specificity (95% and 98%) is shown for the 11 proteins with an AUC > 0.70 in Table 3. At 95% specificity, CA125 had a sensitivity of 0.879 (95% CI: 0.802–0.940) and HE4 had a sensitivity of 0.612 (95% CI: 0.526–0.716). Similarly, at 98% specificity, CA125 ranked first, with a sensitivity of 0.810 (95% CI: 0.707–0897) and HE4 ranked second with a sensitivity of 0.578 (95% CI: 0.302–0.672). When the performance in combination with CA125 was considered, HE4 no longer ranked at the top. Instead, SEZ6L and ITGAV had the highest sensitivities at 98% specificity. 

### 3.4. Development of a Multiprotein Classifier for Early Stage Ovarian Cancer

To improve the detection of ovarian cancer at an early stage over CA125 alone, we used a statistical learning method to develop a multiprotein classifier that could distinguish sera from early stage ovarian cancer patients from that of healthy control women. Using LASSO logistic regression to adaptively perform variable selection, the expression of 68 cancer-related proteins were considered as potential predictors, with the optimal model combining the expression values of CA125 with three additional proteins (HE4, ITGAV, and SEZ6L), such that the predicted ovarian cancer risk score is equal to:

(1) expit(−3.43+0.959× CA125+0.380× HE4+−0.946× ITGAV +−0.964× SEZ6L)

where expit(x) = *e*^x^/(1 + *e*^x^). While this risk score would typically equal the estimated probability of ovarian cancer, the intercept estimate is biased given the case-control study design and, thus, we call it more generally a risk score. The positive weights for CA125 and HE4 indicate a higher predicted likelihood of cancer for those with a higher expression, while the negative weights for ITGAV and SEZ6L indicate a lower predicted likelihood of cancer for those with a higher expression. Interestingly, ITGAV and SEZ6L have not previously been identified as early stage ovarian cancer biomarkers, and the levels of both proteins were significantly decreased in sera from women with early stage ovarian cancer compared to healthy controls (Table 2, Figure 3a, bottom panels). When we examined the t-SNE plots clustered for all 68 proteins (Figure S1b) by the expression of the four proteins included in our multiprotein classifier, we found that the majority of the cancer samples expressed high levels of CA125 and HE4 (Figure S1c,d) and low levels of ITGAV and SEZ6L (Figure S1e,f).

The ROC curves for the multiprotein classifier and each of the four individual proteins included in the classifier are shown in Figure 3b and summarized in Table 4. Compared to CA125 alone, the four-protein classifier improved the AUC from 0.958 (95% CI: 0.928–0.982) to 0.974 (95% CI: 0.949–0.989). The sensitivity at 98% specificity of the multiprotein classifier was also improved from 0.810 (95% CI: 0.707–0.897) for CA125 alone to 0.862 (95% CI: 0.776–0.933) for the multiprotein classifier. The improvement in the AUC by the multiprotein classifier compared to using CA125 alone was statistically significant (*p* = 0.02). The predictive performance by CA125 measured by ELISA was similar to that of CA125 measured by Proseek, with an AUC of 0.959 (95% CI: 0.934–0.979) and sensitivities at 95% and 98% specificity of 0.858 (95% CI: 0.779–0.920) and 0.779 (95% CI: 0.628–0.876), respectively

### 3.5. Validation of the Multiprotein Classifier for Early Stage Ovarian Cancer Using a New Cohort of Early Stage Ovarian Cancer Samples

To validate our multiprotein classifier on an unrelated set of serum samples, 192 early stage ovarian cancer samples and 467 healthy control samples from four different institutions were assembled as Cohort #2 (Table 1). Similar to Cohort #1, the majority of the serum samples were from women with HGSOC (40%), followed by the endometrioid subtype (26%), clear cell carcinoma (19%), and mucinous ovarian cancer (8%).

The NPX values between Cohort #1 and Cohort #2 were normalized using “bridge” samples from Cohort #1 (see Section 2. Materials and Methods). The comparison of the NPX values prior to normalization for the bridge samples (across all proteins) is shown in Figure S2a and a histogram of the protein-specific normalization factors is shown in Figure S2b. The normalization factors for the four proteins of interest were 0.70 (CA125), 1.02 (HE4), 1.98 (ITGAV), and 1.95 (SEZ6L). 

**Figure 4:**
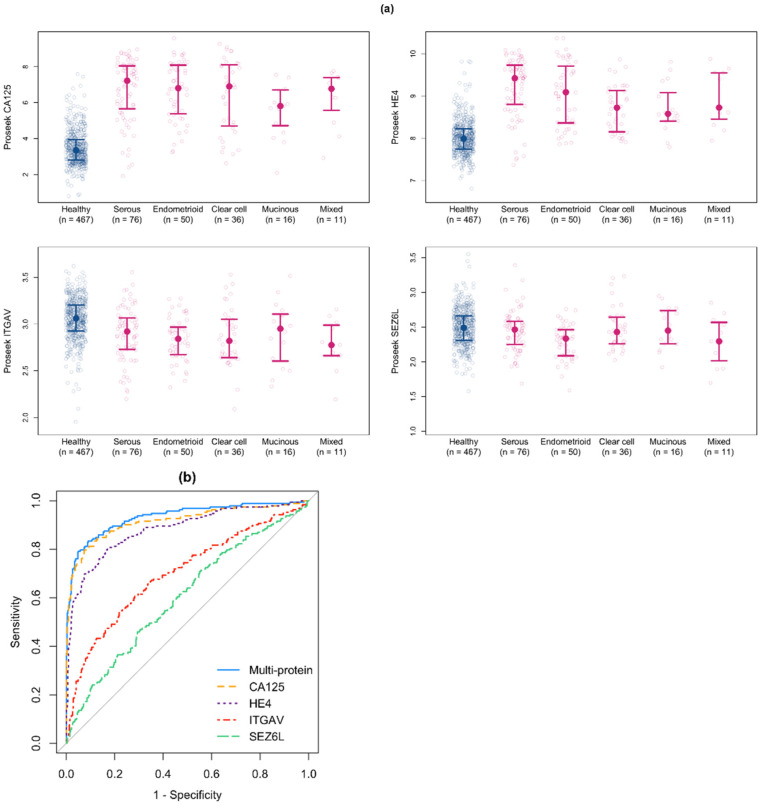
Validation of the multiprotein classifier for use with Cohort #2, a second cohort of early stage ovarian cancer samples. (**a**) Proseek NPX values of the proteins included in the early stage multiprotein classifier between healthy controls and early stage ovarian cancer samples from Cohort #2. The median and 25th/75th percentiles are shown in all plots. (**b**) ROC curves for the early stage multiprotein classifier applied to the Cohort #2 samples and individual proteins included in the early stage multiprotein classifier.

For Cohort #2, we tested all 92 proteins to identify whether they were significantly differentially expressed between the institutions. Of the 24 unstable proteins identified in Cohort #1, 9 proteins (38%) were also unstable in Cohort #2. Of the 68 stable proteins in Cohort #1, only 3 proteins (4%) were found to be unstable in Cohort #2. These three proteins (CA125, MK, and TFPI-2) were overexpressed in the cancer samples for all institutions, but the magnitude of overexpression varied between institutions. For the 15 unstable proteins identified in Cohort #1, but not Cohort #2, there was significant differential expression between Cohort #2 and one of the Cohort #1 institutions (TX). When we compared the NPX values for CA125 to the clinical values for the ovarian cancer patients in Cohort #2, we found a correlation of 0.78, similar to what was observed in Cohort #1. The protein fold changes between ovarian cancer and healthy patients were similar for Cohort #1 and Cohort #2 (Figure S2c). The NPX values in Cohort #2 for the four classifier proteins separated by ovarian cancer subtype are shown in Figure 4a. 

We next applied the early stage multiprotein classifier to the Cohort #2 samples in order to validate its performance. The ROC curve for the early stage multiprotein classifier applied to the Cohort #2 samples, along with the ROC curves for the four proteins individually, are shown in Figure 4b and summarized in Table 4. For the multiprotein classifier, the AUC was 0.933 (95% CI: 0.909–0.955). The sensitivity at 95% specificity was 0.792 (95% CI: 0.708–0.844) and the sensitivity at 98% specificity was 0.661 (95% CI: 0.526–0.771). For CA125 alone, the AUC was 0.916 (95% CI: 0.886–0.942). The modest improvement in AUC by the multiprotein classifier compared to using CA125 alone was statistically significant (*p* < 0.001).

### 3.6. Validation of the Multiprotein Classifier Using Serum Samples from Women with Benign Ovarian Conditions

In order to examine the performance of our classifier in a broader sample set, we applied the early stage multiprotein classifier to samples from women with benign ovarian conditions from the same institutions as Cohort #1 (*n* = 49) and Cohort #2 (*n* = 115). The mean (SD) age of the women with benign ovarian conditions was 56.5 (14.0) years old with a median of 59 and range of 18–84 years old. The majority of the benign ovarian conditions were serous cystadenomas or adenofibromas (*n* = 101), but also included benign cysts (*n* = 30), mucinous cystadenomas (*n* = 10), endometriotic cysts (*n* = 9), and various other benign ovarian conditions (*n* = 14).

**Figure 5:**
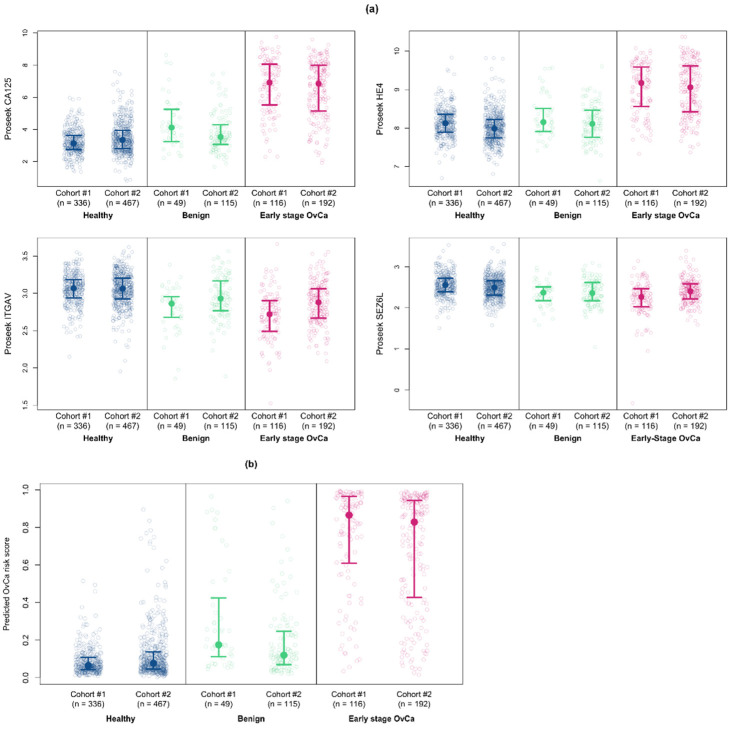
Validation of the multiprotein classifier with serum samples from women with benign ovarian conditions. (**a**) Comparison of the NPX values for the proteins included in the multiprotein classifier between healthy controls, benign samples, and the early stage ovarian cancer samples from both cohorts of samples. (**b**) Predicted cancer risk scores stratified by true cancer status for the early stage ovarian cancer, benign, and healthy control samples from Cohort #1 and Cohort #2. The median and 25th/75th percentiles are shown in all plots.

These serum samples were run on the Proseek Oncology II panel simultaneously with the ovarian cancer and healthy controls. The NPX expression levels for the four proteins in our multiprotein classifier are shown for ovarian cancer, benign, and healthy control samples from both cohorts in Figure 5a. In general, the median NPX values for the benign samples were intermediate between those of the healthy controls and the ovarian cancer samples or similar to the NPX values from the healthy controls. The predicted cancer risk scores stratified by true cancer status are shown in Figure 5b for both cohorts. Using the 98% specificity cutpoint, our multiprotein classifier correctly classified 80.5% of benign samples as “not cancer”. 

### 3.7. Validation of the Multiprotein Classifier for Early Stage Ovarian Cancer on Samples from Women with Late Stage Ovarian Cancer

**Figure 6:**
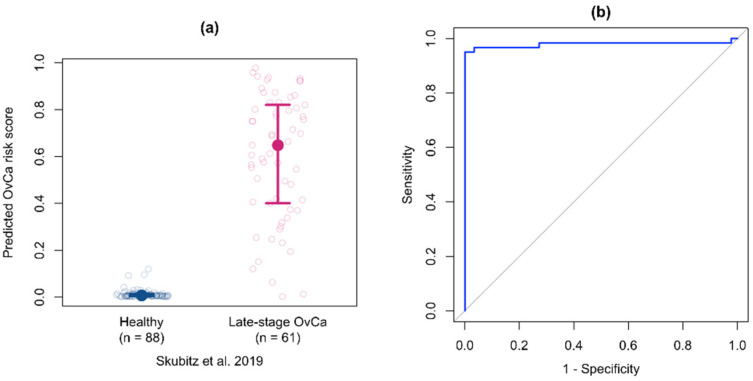
Validation of the multiprotein classifier with serum samples from women with late stage ovarian cancer. (**a**) Predicted cancer risk scores with median and 25th and 75th percentiles stratified by true cancer status for late stage ovarian cancer samples (*n* = 61 cancer samples; *n* = 88 healthy controls) from Skubitz et al. [12]. (**b**) ROC curve for the early stage multiprotein classifier applied to the late stage samples.

In a previous study, we tested sera from women with late stage high grade serous ovarian cancer and healthy women on the Proseek Oncology II panel [12]. However, it has been suggested that protein changes in early stage disease may not persist to later stage. To determine if the multiprotein classifier developed using the early stage samples from Cohort #1 could retain its performance if presented with late stage samples, we applied the early stage multiprotein classifier to the NPX data from late stage samples. Similar to what we observed in the early stage samples, CA125 and HE4 levels were elevated in the late stage ovarian cancer samples, while ITGAV and SEZ6L levels were higher in the healthy control samples. The predicted cancer risk scores, stratified by true cancer status, are shown in Figure 6a for the late stage samples. Given the lack of bridge samples between these two studies, we were unable to normalize between experiments, and thus the risk scores are not directly comparable between studies. However, we can see the multiprotein risk score discriminates the late stage samples from the healthy controls. For the ROC curve for the late-stage samples using the early stage multiprotein classifier (Figure 6b), the AUC was 0.978 (95% CI: 0.941–1.00). The sensitivity at 95% specificity was 0.967 (95% CI: 0.902–1.00) and the sensitivity at 98% specificity was 0.951 (95% CI: 0.885–1.00). These data demonstrate that the multiprotein classifier developed using early stage samples has strong discrimination performance between healthy and late stage ovarian cancer samples. 

## 4. Discussion

In this study, we used the Proseek Oncology II panel to sensitively quantify the level of proteins in sera from early stage ovarian cancer patients and healthy controls, with the goal of developing and testing a multiprotein classifier for the detection of ovarian cancer at an early stage of disease when it is more treatable. The Proseek Oncology II panel uses PEA technology to quantify 92 different cancer-related proteins, including the well-known ovarian cancer serum biomarkers CA125 and HE4. By analyzing data from a cohort of 116 early stage ovarian cancer and 336 healthy control patients from two institutions, we developed a multiprotein classifier to distinguish ovarian cancer cases from healthy controls. Our classifier was comprised of four proteins: CA125, HE4, ITGAV, and SEZ6L. When we tested our four-protein classifier with a validation cohort comprised of 192 early stage ovarian cancer and 467 healthy control patients from four different institutions, we found that it performed significantly better than CA125 alone. 

Of the 27 proteins that were significantly differentially expressed in both cohorts of serum, 11 proteins were found at decreased levels in ovarian cancer samples compared to the healthy controls, including the two proteins in our multiprotein classifier, ITGAV and SEZ6L. ITGAV is a subunit of the alpha V family of integrins, that are involved in cell–cell and cell–matrix adhesions and signaling [36]. High expression of ITGAV in ovarian cancer tumor tissue from late stage tumors has been associated with poor prognosis [37]. However, both tissue [38] and serum levels of ITGAV have been shown to be present at reduced levels in ovarian cancer compared to benign tumors and borderline ovarian cancers [39]. In addition, ITGAV expression has been correlated with increased expression of the matrix metalloprotease MMP9 in ovarian cancer effusions [40], which could impact ITGAV shedding into the serum in late stage ovarian cancer. The SEZ6L protein is a single pass transmembrane protein that may contribute to specialized endoplasmic reticulum functions [41]. Genetic analyses have implicated the loss of SEZ6L gene function in the risk for development of lung cancer by deletion [42], and in colon cancer through promoter hypermethylation [43]. Although, Gorlov et al. also showed increased expression of SEZ6L in lung cancer cell lines and tumor tissues compared to normal lung cells, suggesting that SEZ6L is both a tumor biomarker and a genetic risk factor [42]. 

In our previous analysis of the Proseek Oncology II biomarker panel, 40 proteins were found to have significantly lower levels in sera from women with late stage HGSOC [12]. Eight of the eleven proteins with significantly lower levels in early stage ovarian cancer were also found at reduced levels in sera from late stage HGSOC, including ITGAV [12], while three biomarkers (CPE, IFNg-R1, SEZ6L) were significantly lower only in the early stage samples 

While the identification of protein biomarkers at lower levels in cancer compared to normal sera seems somewhat counter-intuitive, others have shown similar results for SCF [44] and FASL [45]. One explanation could be that lower levels of proteins involved in immune response could result in reduced antitumor immunity in ovarian cancer patients, as was also suggested by Arts et al. for FASL [45]. Indeed, seven of the eleven proteins that we showed had lower levels in sera from early stage ovarian cancer patients than in the healthy controls play a role in immune response [41]. Alternatively, antigen–autoantibody complex formation could mask the epitopes recognized by the Proseek assay, causing lower levels of protein to be detected in ovarian cancer patients. Another explanation could be that the proteins found at lower levels in ovarian cancer sera are more actively cleared and/or catabolized by the tumor-bearing host. However, for proteins present at very low levels in all samples, the difference may simply reflect the high degree of heterogeneity within the population [46], which can only be revealed by the inclusion of a large number of healthy control samples in the study. In our discovery cohort, we included a 3:1 ratio of control to ovarian cancer samples in an attempt to address this issue. However, given the relatively low prevalence of ovarian cancer in the population, the inclusion of even more control samples would likely improve classifier performance. 

Panels of biomarkers, such as ours, with greater sensitivity than CA125 alone could increase the proportion of early stage cancers detected, possibly translating to improved mortality. The recent report of the mortality results of the UKCTOCS screening trial [47] casts some doubt on the assumption that earlier detection of ovarian cancer will improve rates of cure. In this large ovarian cancer screening trial, multimodal screening (CA125 and transvaginal sonography) detected 10% more early stage (I-II) and fewer late-stage (III-IV) ovarian cancers, though this did not result in improved mortality [47]. In the NROSS trial in the United States, a similar strategy with CA125 and transvaginal sonography produced a 41% stage shift [48]. As the NROSS study was not adequately powered or controlled to assess mortality, it is not possible to determine whether the much greater stage shift would be associated with significantly decreased mortality. Panels of biomarkers with greater sensitivity could, however, further increase the fraction of early stage cancers detected, prompting additional trials to assess the impact of screening on mortality.

Although we developed our multiprotein classifier using early stage ovarian cancer samples compared to healthy controls, serum samples from women with benign ovarian conditions were run simultaneously on each of the Proseek Oncology II plates. When we applied our multiprotein classifier to the 164 benign samples with a threshold of 98% specificity, 80.5% of the benign samples were classified correctly, with only ~20% of the benign cases being classified as “cancer”. Our multiprotein classifier could be incorporated into a two-step screening strategy, such as those used in the UKCTOCS and NROSS studies [47,48], whereby those women whose serum tests indicate “cancer”, would then be screened by imaging to rule out the false–positive benign lesions and exclude them from surgery. 

Other groups have used multiple panels of the Proseek multiplex assays (Oncology II, Inflammation, Immune Response, Development, Cell Regulation, Metabolism, Cardiometabolic, and Organ Development) to identify biomarkers in cohorts of ovarian cancer patients compared to benign and borderline cases [39,49] and healthy controls (Oncology II, Inflammation, Neurology, CVD II and CDV III, Ref. [50]) and have developed multiprotein classifiers from their data. As with our classifier, these all included both CA125 and HE4, while some classifiers included up to 13 additional biomarkers from the 460 proteins assayed [50]. Most of the biomarkers included in these multiprotein classifiers were elevated in ovarian cancer compared to benign, borderline, and control samples. However, in a recent analysis of 177 Proseek proteins (from the Oncology II and Inflammation panels) used to distinguish benign tumors from borderline tumors and ovarian cancer, ITGAV was the only individual biomarker found to improve the performance of the reference model (CA125, HE4 and age) [39]. ITGAV was also included in the six-protein classifier developed using LASSO regression, and was one of only two proteins to have significantly lower NPX values in the ovarian cancer samples compared to benign samples [39]. 

Recently, Gyllensten et al. used the Olink Explore platform to analyze 1493 proteins in plasma samples from women with ovarian cancer compared to benign ovarian tumors [51]. They identified 28 proteins with significantly elevated levels in two small cohorts of samples and developed several multiprotein models which were able to distinguish ovarian cancer from benign samples. The models used between four–seven proteins, including HE4, but interestingly, none of the models included CA125 [51]. 

Another recent study used the Proseek Oncology II panel to identify biomarkers capable of distinguishing ovarian cancer cases in prediagnostic samples from the European Prospective Investigation into Cancer and Nutrition (EPIC) cohort [52]. They identified nine individual biomarker proteins that could discriminate between 91 women who later developed cancer (39 women <9 months; 52 women <18 months) from 182 women who did not, with an AUC of ≥0.70. Seven of these nine proteins (CA125, HE4, CXCL13, FOLR1, KLK11, MK and MSLN) were significantly elevated in both of our early stage ovarian cancer cohorts. In their study, the addition of any of the individual markers to CA125 did not significantly improve the ability to detect the prediagnostic samples, and the use of all 92 markers to develop a detection algorithm was also not successful. In contrast, our classification algorithm included CA125, HE4, and two markers with lower expression in the early stage ovarian cancer samples.

The Proseek Oncology II panel uses PEA technology to provide relative quantification of 92 different cancer-related proteins in serum or plasma. As was done in our study, “bridge” samples are necessary to compare results between different experiments due to the relative quantification. For clinical application, absolute quantification of proteins would be necessary. Additionally, in our analysis of sera from women with early stage ovarian cancer, we found that 24 of the 92 proteins differed significantly between ovarian cancer cases and control samples that were collected from two different institutions in Cohort #1. This data suggests that these 24 proteins may have unstable levels due to their sensitivity to preanalytical variation. Indeed, of the nine proteins in our study that overlapped with the analysis of preanalytical variables in the Proseek Cardiovascular Disease I and Inflammation I assay panels done by Shen et al. [13], the four markers that we found had significant *p*-values for differential effects by institution were also shown to have significantly increased ΔNPX values with increasing time from blood collection to processing [13]. The increase in protein levels with increasing time to centrifugation could be attributed to protein leakage from blood cells. In Shen et al.’s analysis [13], only minimal effects on the protein profiles were observed after an 8 h delay in centrifugation (at 4 degrees) and 1 h at room temperature. However, after 24 h at room temperature, nearly one-third of the proteins were affected [13]. In our study, more than 25% of the measured proteins showed significant differential effects by institution, suggesting a potential for preanalytical variation in sample processing, despite the use of standardized methods for sample processing and timely storage. This underlines the necessity of selecting robust biomarker candidates for clinical assay development. The instability of biomarkers in serum may explain the inconsistent results from the literature; for example, EGF has been reported to be elevated [53], decreased [54], or not significantly different [55] in sera from ovarian cancer patients compared to healthy controls. Interestingly, in the study by Shen et al., essentially no change was observed in protein levels quantified by the Proseek assay after up to eight freeze–thaw cycles [13], indicating that multiple rounds of freezing and thawing does not lead to destabilization of these proteins. Other studies have shown differences in biomarker levels due to long term storage [56], common medications [57], and samples collected under anesthesia [58].

Thus, although several studies have used the Olink platform in an effort to identify ovarian cancer biomarkers, slightly different panels of biomarkers have been found. In terms of prioritizing a biomarker panel for future study, it would be useful to further characterize the performance of existing panels on external validation samples in settings where the panel’s biomarkers have already been quantified in samples from other studies. Following that, prospective validation of the most promising panels—or a combination of panels—would be needed to determine if the early detection of ovarian cancer is increased, and whether there is an associated mortality reduction.

## 5. Conclusions

Our study highlights the importance of identifying protein biomarkers that are robust and not sensitive to preanalytical variation. We also showed that including protein biomarkers that are present at reduced levels in early stage ovarian cancer cases compared to healthy controls can increase the predictive value of a multiprotein classifier, suggesting that the lower levels of some proteins may contribute to tumor development. Future models may need to include autoantibodies, circulating tumor DNA, miRNA, or other molecules. In addition, longitudinal testing of protein panels and other biomarkers may be necessary to improve the earlier detection of ovarian cancer.

## Figures and Tables

**Table 1:** Patient demographic information for Cohorts #1 and #2.

	Cohort #1 (Discovery)		Cohort #2 (Validation)	
	Healthy (*n* = 336)	Early Stage Ovarian Cancer (*n* = 116)	Healthy (*n* = 467)	Early Stage Ovarian Cancer (*n* = 192)
Location				
MN	61 (18%)	46 (40%)		
TX	275 (82%)	70 (60%)		
Fox Chase			226 (48%)	55 (29%)
Italy—Milan			144 (31%)	86 (45%)
OHSU			7 (1%)	12 (6%)
BWH-Harvard			90 (19%)	39 (20%)
Age (years)				
Mean (SD)	66.4 (7.6)	58.5 (12.5)	55.1 (11.5)	56.3 (11.5)
Median	67.0	58.5	54.0	56.0
Range	48–87	19–85	24–85	24–85
CA125 value				
Median (Q1, Q3)	10.4 (7.7, 14.3)	98.3 (42.9, 379)	ND	143 (42.3, 543)
Range	0–69	7–22,780	ND	2–12,219
Subtype				
HGSOC		53 (46%)		76 (40%)
Endometrioid		21 (18%)		50 (26%)
Clear cell		14 (12%)		36 (19%)
Mucinous		15 (13%)		16 (8%)
Mixed		13 (11%)		11 (6%)
Other		0 (0%)		3 (2%)
Stage				
I		73 (63%)		119 (62%)
II		43 (37%)		73 (38%)

Abbreviations: MN (University of Minnesota), TX (M.D. Anderson), OHSU (Oregon Health & Science University), BWH (Brigham Women’s Hospital), SD (standard deviation), Q1 (first quartile), Q3 (third quartile), ND (not done), HGSOC (High-Grade Serous Ovarian Cancer).

**Table 2:** Mean (standard deviation) of NPX values for the 38 proteins in Cohort #1 significantly different between ovarian cancer and healthy controls.

Protein	UniProt ID	Healthy (*n* = 336)	Early Stage Ovarian Cancer (*n* = 116)	*p*-Value
CA125	Q8WXI7	3.21 (0.77)	6.73 (1.68)	<0.001
HE4	Q14508	8.14 (0.39)	9.02 (0.65)	<0.001
ITGAV	P06756	3.05 (0.21)	2.70 (0.32)	<0.001
MK	P21741	6.40 (0.60)	7.17 (0.95)	<0.001
SCF	P21583	8.89 (0.41)	8.22 (0.85)	<0.001
IL6	P05231	2.89 (1.26)	4.21 (1.68)	<0.001
SEZ6L	Q9BYH1	2.56 (0.27)	2.22 (0.44)	<0.001
FASLG	P48023	8.97 (0.48)	8.58 (0.52)	<0.001
ESM-1	Q9NQ30	8.98 (0.57)	9.44 (0.62)	<0.001
hK11	Q9UBX7	6.18 (0.44)	6.74 (0.86)	<0.001
ADAM-TS 15	Q8TE58	1.86 (0.63)	2.41 (0.90)	<0.001
XPNPEP2	O43895	8.06 (0.58)	7.61 (0.73)	<0.001
SYND1	P18827	6.06 (0.50)	6.51 (0.82)	<0.001
CXCL13	O43927	7.66 (0.61)	8.14 (0.86)	<0.001
TFPI-2	P48307	7.57 (0.49)	7.98 (0.76)	<0.001
TCL1A	P56279	4.01 (1.22)	3.28 (1.31)	<0.001
FR-α	P15328	6.57 (0.48)	7.12 (1.08)	<0.001
KLK13	Q9UKR3	3.41 (0.75)	3.84 (0.87)	<0.001
VEGFR-2	P35968	6.70 (0.28)	6.56 (0.30)	<0.001
CEACAM1	P13688	6.02 (0.24)	5.91 (0.25)	<0.001
TLR3	O15455	4.93 (0.67)	4.56 (0.87)	<0.001
MSLN	Q13421	3.12 (0.66)	3.55 (1.03)	<0.001
CYR61	O00622	5.70 (0.49)	5.37 (0.83)	0.001
GPNMB	Q14956	6.07 (0.19)	5.97 (0.24)	0.001
CPE	P16870	3.95 (0.42)	3.72 (0.58)	0.002
LY9	Q9HBG7	5.17 (0.41)	4.96 (0.53)	0.003
NECT4	Q96NY8	4.03 (0.47)	4.36 (0.92)	0.004
ERBB2	P04626	7.44 (0.31)	7.27 (0.49)	0.004
TNFRSF6B	O95407	5.10 (0.78)	5.51 (1.13)	0.005
FCRLB	Q6BAA4	0.92 (0.52)	1.18 (0.74)	0.01
GPC1	P35052	4.64 (0.39)	4.44 (0.55)	0.01
IFN-γ-R1	P15260	4.68 (0.32)	4.53 (0.43)	0.01
CD48	P09326	5.86 (0.32)	5.73 (0.42)	0.01
RET	P07949	5.35 (0.48)	5.12 (0.67)	0.01
ICOSLG	O75144	5.94 (0.57)	5.71 (0.73)	0.03
CTSV	O60911	3.74 (0.48)	3.54 (0.64)	0.03
AREG	P15514	1.87 (0.57)	2.07 (0.62)	0.03
MIA	Q16674	9.66 (0.29)	9.55 (0.37)	0.03

**Table 3:** AUC and sensitivities at 95% and 98% specificity for the 11 proteins with AUC > 0.70 comparing women with ovarian cancer to healthy women in Cohort #1. Summaries are also given for the proteins when combined with CA125.

	Single-Protein AUC		Single-Protein Sensitivity at 95% Specificity		Single-Protein Sensitivity at 98% Specificity	
Protein	Estimate (95% CI)	Rank	Estimate (95% CI)	Rank	Estimate (95% CI)	Rank
CA125	0.958 (0.928, 0.982)	1	0.879 (0.802, 0.940)	1	0.810 (0.707, 0.897)	1
HE4	0.857 (0.808, 0.901)	2	0.612 (0.526, 0.716)	2	0.578 (0.302, 0.672)	2
ITGAV	0.832 (0.783, 0.878)	3	0.440 (0.302, 0.621)	3	0.276 (0.164, 0.440)	4
SCF	0.778 (0.728, 0.825)	4	0.336 (0.233, 0.448)	6	0.293 (0.172, 0.371)	3
SEZ6L	0.764 (0.709, 0.816)	5	0.310 (0.207, 0.448)	9	0.164 (0.078, 0.310)	14
IL6	0.762 (0.708, 0.812)	6	0.310 (0.129, 0.474)	9	0.129 (0.000, 0.233)	22
MK	0.745 (0.685, 0.802)	7	0.405 (0.310, 0.509)	4	0.250 (0.052, 0.431)	6
ESM-1	0.718 (0.661, 0.772)	8	0.250 (0.129, 0.353)	17	0.121 (0.060, 0.224)	28
hK11	0.713 (0.651, 0.770)	9	0.353 (0.224, 0.466)	5	0.216 (0.138, 0.336)	9
ADAM-TS 15	0.710 (0.649, 0.768)	10	0.259 (0.181, 0.379)	16	0.233 (0.129, 0.310)	8
FASLG	0.705 (0.647, 0.761)	11	0.284 (0.172, 0.414)	12	0.181 (0.026, 0.267)	12
	**Protein + CA125 AUC**		**Protein + CA125 Sensitivity** **at 95% Specificity**		**Protein + CA125 Sensitivity** **at 98% Specificity**	
**Protein**	**Estimate (95% CI)**	**Rank**	**Estimate (95% CI)**	**Rank**	**Estimate (95% CI)**	**Rank**
CA125	--	--	--	--	--	--
HE4	0.966 (0.944, 0.983)	8	0.853 (0.784, 0.914)	55	0.784 (0.664, 0.888)	55
ITGAV	0.967 (0.941, 0.987)	7	0.914 (0.845, 0.957)	1	0.862 (0.776, 0.931)	2
SCF	0.958 (0.927, 0.982)	46	0.879 (0.802, 0.940)	36	0.810 (0.707, 0.897)	33
SEZ6L	0.974 (0.950, 0.992)	1	0.905 (0.845, 0.957)	3	0.897 (0.836, 0.948)	1
IL6	0.963 (0.935, 0.983)	15	0.836 (0.759, 0.914)	61	0.793 (0.707, 0.862)	51
MK	0.959 (0.931, 0.982)	29	0.862 (0.776, 0.931)	52	0.784 (0.698, 0.871)	55
ESM-1	0.960 (0.931, 0.983)	24	0.862 (0.802, 0.940)	52	0.802 (0.716, 0.897)	45
hK11	0.953 (0.923, 0.976)	65	0.828 (0.750, 0.914)	66	0.776 (0.672, 0.853)	60
ADAM-TS 15	0.961 (0.931, 0.984)	21	0.888 (0.810, 0.940)	22	0.793 (0.716, 0.905)	51
FASLG	0.973 (0.954, 0.988)	2	0.914 (0.853, 0.974)	1	0.836 (0.733, 0.931)	11

Abbreviations: AUC (area under the curve), CI (confidence interval).

**Table 4:** Area under the curve (AUC) and sensitivities at 95% and 98% specificity (with 95% confidence intervals (CIs)) for the multiprotein classifier and the individual protein components for Cohorts #1 and #2.

	AUC	Sensitivity at 95% Specificity	Sensitivity at 98% Specificity	*p*-Value ^1^
Cohort #1				
Multiprotein	0.974 (0.949, 0.989)	0.914 (0.852, 0.964)	0.862 (0.776, 0.933)	--
CA125	0.958 (0.928, 0.982)	0.879 (0.802, 0.940)	0.810 (0.707, 0.897)	0.02
HE4	0.857 (0.808, 0.901)	0.612 (0.526, 0.716)	0.578 (0.302, 0.672)	<0.001
ITGAV	0.832 (0.783, 0.878)	0.440 (0.302, 0.621)	0.276 (0.164, 0.440)	<0.001
SEZ6L	0.764 (0.709, 0.816)	0.310 (0.207, 0.448)	0.164 (0.078, 0.310)	<0.001
Cohort #2				
Multiprotein	0.933 (0.909, 0.955)	0.792 (0.708, 0.844)	0.661 (0.526, 0.771)	--
CA125	0.916 (0.886, 0.942)	0.745 (0.672, 0.807)	0.635 (0.536, 0.750)	<0.001
HE4	0.882 (0.850, 0.912)	0.620 (0.536, 0.703)	0.516 (0.359, 0.630)	<0.001
ITGAV	0.700 (0.653, 0.746)	0.266 (0.172, 0.354)	0.109 (0.036, 0.224)	<0.001
SEZ6L	0.603 (0.554, 0.651)	0.130 (0.068, 0.182)	0.057 (0.016, 0.115)	<0.001

^1^ Comparing the AUC of the multiprotein classifier to the AUC of the listed classifier.

## Data Availability

Data available upon reasonable request.

## Supplementary Materials

The following supporting information can be downloaded at: https://www.mdpi.com/article/10.3390/cancers14133077/s1, Figure S1: PCA and t-SNE plots of 452 serum samples clustered by 67 proteins; Figure S2: Normalization of Cohort #2 using “bridge” samples from Cohort #1 and comparison of protein-specific fold changes between cohorts; Table S1: The mean differences in protein levels between cancer and healthy samples by institution for the 24 proteins with differential expression by institution for Cohort #1; Table S2: Mean (SD) of NPX values for 68 proteins in Cohort #1; Table S3: AUC and sensitivities at 95% and 98% specificity for single proteins and proteins in combination with CA125 when comparing women with ovarian cancer to healthy women in Cohort #1.
