# Supplementary material for: Development of a Multiprotein Classifier for the Detection of Early Stage Ovarian Cancer
Source: Cancers (Basel). 2022 Jun 23;14(13):3077. doi: 10.3390/cancers14133077 (PMC9264950; doi:10.3390/cancers14133077)
Supplement: Supplementary file 1 [file cancers-14-03077-s001.zip › cancers-1755075-supplementary/Figures S1-S2.pdf]

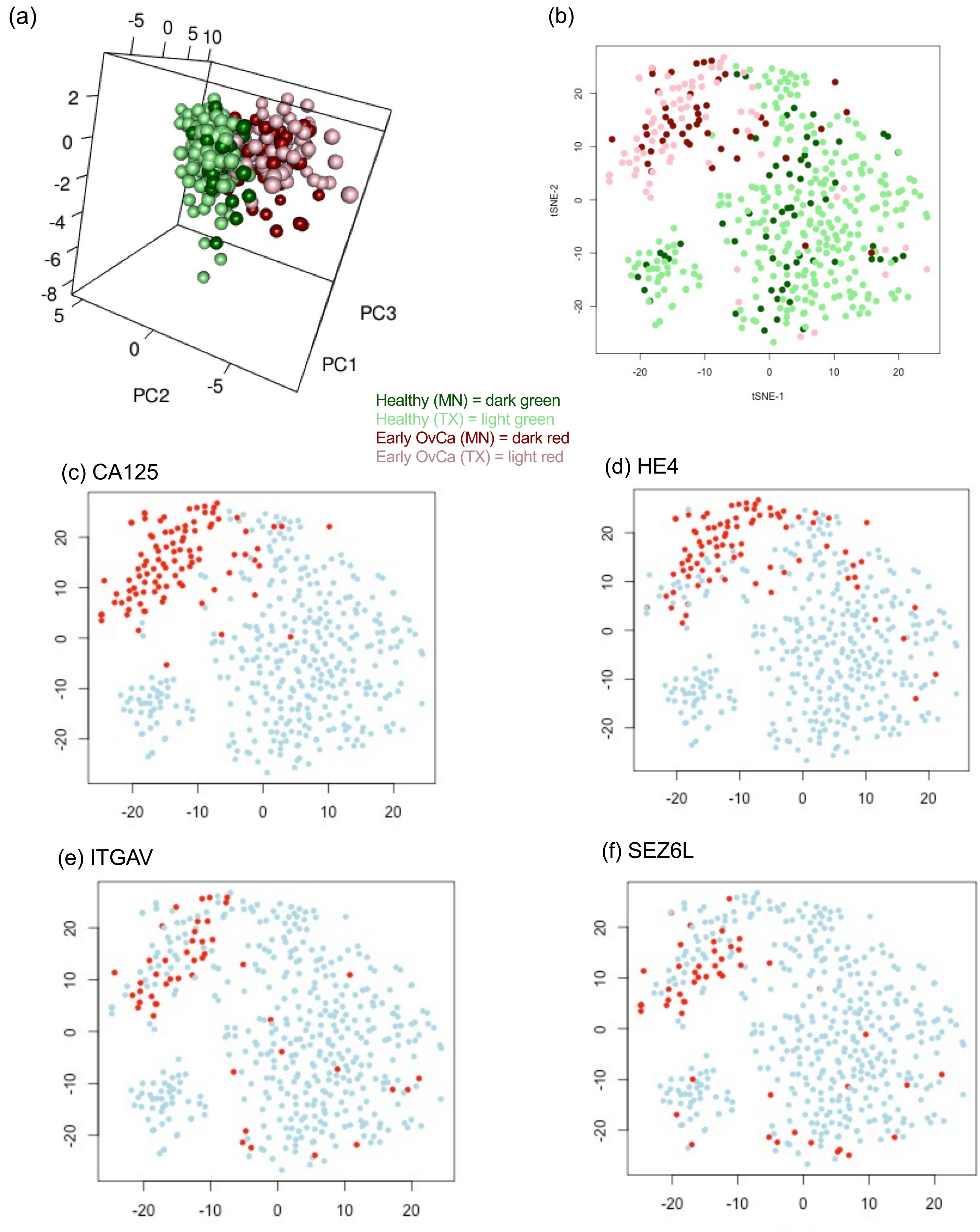

Supplementary Figure S1. Plots of 452 serum samples clustered by 67 proteins. PCA (a) and t-SNE plots (b) show that the 116 early stage ovarian cancer samples (red; clustered in the upper left corner of the plot) separate from the 336 healthy control samples (green) in Cohort #1. (c-f) t-SNE plots colored by protein levels: (c) CA125 ELISA values > 35 (5.13 log2) = red, (d) HE4 > 8.8 = red, (e) ITGAV < 2.6 = red, and (f) SEZ6L < 2.1 = red.

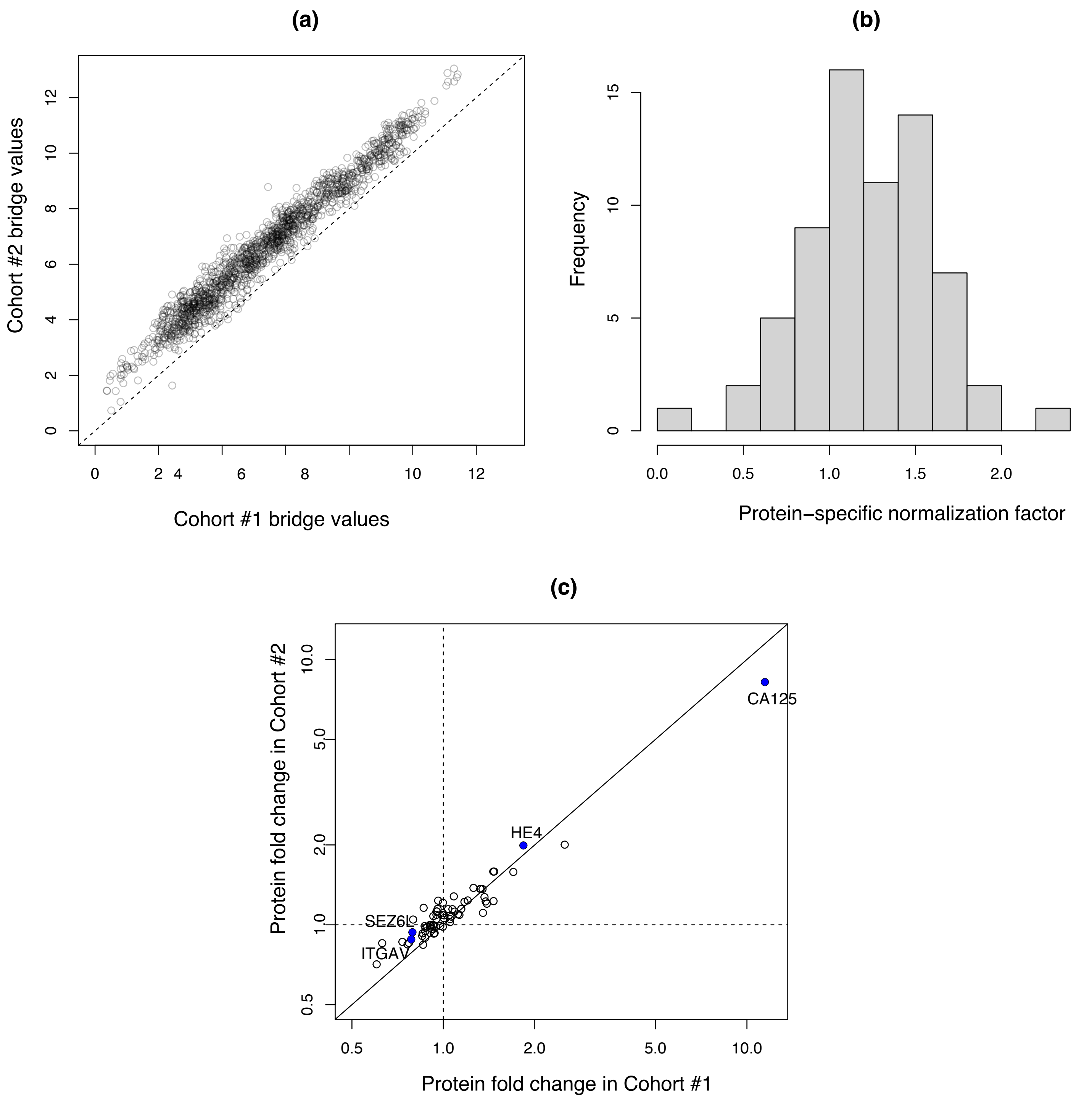

Supplementary Figure S2. Normalization of Cohort #2 using “bridge” samples from Cohort #1. (a) Prior to normalization, the association between the NPX values from Cohort #1 vs. Cohort #2 for the 22 bridge samples across all 68 proteins. (b) The protein-specific normalization factors for the 68 proteins. (c) Protein fold changes in Cohort #1 vs. Cohort #2.
